# “When I need help, I ask my friends”: experiences of Spanish autistic women when disclosing their late diagnosis to family and friends

**DOI:** 10.3389/fpsyg.2024.1342352

**Published:** 2024-04-23

**Authors:** Irene Garcia-Molina

**Affiliations:** Department of Developmental, Educational, Social Psychology and Methodology, Institute of Feminist and Gender Studies, Universitat Jaume I, Castellón, Spain

**Keywords:** autism, autistic women, disclosure/disclose, late diagnosis, friendship, family

## Abstract

Family and friends may play an important role both in the identification and diagnosis of any condition, as well as in the provision of support afterwards. However, when the diagnosis is autism and it arrives late, as is often the case with autistic women, we find the double stigma of experiencing the repercussions of a late diagnosis, along with the disbelief of those closest to them. This study aims to analyse and understand the experiences of autistic women with their family and friends throughout the diagnostic process and subsequent help-seeking endeavors. A total of 21 Spanish autistic women aged between 20 and 58 years answered a series of open-ended questions—respecting the participants' preferred modality. From the thematic analysis, two main themes emerged regarding the reactions of family and friends, dividing the experiences into two temporal phases: when disclosing the diagnosis and sometime later. Most participants noted that their relatives did not believe them, reacted in a hostile way, or did not give it any importance at the time of disclosure. As a result, their relationship deteriorated even further over time. In contrast, their friends served as pillars of support from the beginning, along with their partners and the associations they attended, because of which they met more autistic women who became their “family”. Thus, another consequence of late diagnosis in autistic women is highlighted, the incomprehension or disbelief from their relatives, and the important role of friendships among women and among autistic women.

## 1 Introduction

A delay in the diagnosis of any condition can have devastating consequences for the person and even more so when they do not find the necessary support within their close circle (family, friends, and co-workers), which is the reality for many autistic women (identity-first language is used in this manuscript, following Kenny et al., 2016). On the one hand, they face underdiagnosis, misdiagnosis, or late diagnosis—which can cause frustration at individual and societal levels as well as health and mental health problems derived from incorrect intervention or even an absence thereof (Rynkiewicz et al., 2019). On the other hand, autistic women also have to deal with the reactions of their environment when they disclose their condition, akin to “coming out of the closet” about being openly autistic, once the diagnosis happens (García-Molina and Fernández-Camahort, 2023). When a diagnosis is disclosed for the first time, it not only requires effort and preparation by the discloser but also a certain level of empathy from the receiver, who might be oblivious to the possible condition of their relative or friend. This phenomenon can be referred to as the double stigma of autistic women, i.e., receiving a late diagnosis and not being believed by her close circle. To the author's knowledge, recent research offers a viewpoint of positive discourse toward autism (Richards, 2016), and there is a growing interest in understanding autistic adults' perspectives about disclosing their autistic identity (Farsinejad et al., 2022). However, there is no known study (especially in the Spanish population) about how family and friends react when autistic women decide to disclose their diagnosis in adulthood.

It is beginning to be widely recognized that autistic women have learned to voluntarily and involuntarily camouflage themselves, masking their differences to fit in socially, which they also tend to do during the administration of differential tests for their diagnosis (Cook et al., 2021; Halsall et al., 2021; Chmia-Torrecillas, 2022). However, there has been limited focus on how they go unnoticed within their own household, or, rather, in that of their parents (or family nucleus), since often it is not until adulthood that the diagnosis happens (Green et al., 2019), when many have already formed their own families (Garcia-Molina and Cortés-Calvo, 2024). In addition to all these challenges, once they have received the diagnosis in adulthood, they must decide whether or not to disclose it to their closest friends, who may hold beliefs and misconceptions about autism. Critically, as highlighted by Davidson and Henderson (2010), “safe people” (for disclosure) may not always those within the close family circle or social relations.

Evidence indicates that many autistic people (adolescents and adults) forgo disclosing their diagnosis due to the perception of potential negative consequences, discrimination, and stigma (Cage and Troxell-Whitman, 2020; Thompson-Hodgetts et al., 2020; Farsinejad et al., 2022). Huang et al. (2022) analyzed the disclosure experiences of 393 autistic people (aged 17–83 years). Most participants in their longitudinal surveys disclosed their diagnosis to someone, usually a friend. Among participants who were diagnosed when they were minors, 71.6% had disclosed it to their school. Furthermore, men were more likely to have disclosed to the school than women; however, since many of these girls were diagnosed at an early age, questions about disclosure to family and partner/spouse were omitted in a high percentage of the participants. Of those who were working or studying, 64.1% and 57.8%, respectively, had disclosed this information in their workplace and university, citing reasons for concealment such as potential discrimination, stigma, fear, or negative repercussions. It should be noted that, in general, much of this research has been conducted in educational or work environments (i.e., Johnson and Joshi, 2016; Frost et al., 2019; Romualdez et al., 2021a,b; Huang et al., 2022), and little attention has been paid to disclosure outside of these contexts, such as the more specific case of family and friends (Togher and Jay, 2023).

One of the reasons for this void in the family environment is derived from the fact that most research on family and autism focuses on children, e.g., the impact on the family of having an autistic child (Patel et al., 2022), or even how providers can predict parental reactions and adjust their feedback (Anderberg and South, 2021). During childhood, the diagnosis is initiated and received by the parents or caretakers along with their daughter (it is not the girl who has to disclose it to her family). However, when a woman receives the diagnosis in adulthood, the family becomes another context for disclosure.

Recently, the friends of autistic women have been receiving more attention. As adults, although some autistic women report social difficulties (Bargiela et al., 2016), many others identify friendships and support relationships as key to their success (Webster and Garvis, 2017) and generally feel satisfied with their social lives (Baldwin and Costley, 2016). Compared with non-autistic women, who may have a wider group of friends, autistic women would have one or two best friends, with a very intense relationship, with whom they spend much or all of their time, both physically and virtually. These friends, as they themselves describe, are “those that they trust”, as “they would be there for them” (Sedgewick et al., 2019; Garcia-Molina, 2022). In addition, as a result of such trust and relationship, these friends would be aware of their condition and even warn and help them to deal with day-to-day life activities and prevent their social exclusion (Dean et al., 2014; Garcia-Molina, 2022). Similarly, in the study of Frost et al. (2019) both male and female participants reported that it was rare for them to come out of the closet; however, when they did, they disclosed it to their close friends and long-term romantic partners with the intention of being understood, as well as school personnel for the purpose of obtaining accommodation. However, it should be noted that this study focused on the educational environment (university) and that only four women participated (vs. 14 men and 1 non-binary individual).

As evident from the above discussion, there are limited studies that focus specifically on how autistic women perceive disclosure reactions. In addition, this would be one of the first studies that focus not only on disclosure to friends but also to family. This study aims to analyse and understand the experiences of Spanish autistic women with late diagnosis regarding the reactions of family and friends when disclosing to them about their diagnosis and their participation in the subsequent search for support or as a part of that support.

## 2 Method

### 2.1 Participants

A total of 21 autistic women (aged 20–58 years; *M* = 34.6, *SD* = 11.54) participated and met the following criteria: Participants (a) who identify as female, (b) who have a formal diagnosis on the autism spectrum; (c) with late diagnosis; (d) who are over 18 years of age; and (e) who speak Spanish. The age at diagnosis ranged between 17 and 55 years (*M* = 32.4, *SD* = 10.79). The majority of the participants, seven women, reported living with their parents and siblings; six women with their partner and child/children; two women with their partner; one woman with her children; one woman with her mother; another one with her grandmother; and three women lived alone (one of them lived with her dog).

### 2.2 Measures

The questions used for this research were drawn up by the principal investigator of the Autistic Women project via a process of consultation with autistic women, expert clinicians, master's students, and researchers. Thus, the topics reflected research aims, previous research, clinical insights, and the priorities of members of the autism community (Gowen et al., 2019). The questionnaire/interview consisted of 30 questions, including 15 questions related to family and friendships: “[when you were seeking the diagnosis] did someone [family member, friends...] help you?” or “What was the reaction of your family/friends when they found out about your diagnosis?” (depending on the preferred modality to express themselves).

The responses were all open-ended, and participants could verbalize and explain their experiences with no time or word limits. In addition, all answers were analyzed to detect further experiences regarding family and friend environments from other types of questions. A copy of the interview schedule is available from the author upon request.

### 2.3 Process

Ethical approval for this research was granted by the Ethics Commission of the Universitat Jaume I. The participants were contacted through five associations that agreed to participate in the study. These organizations gave their informed consent via e-mail, and the first contact with the participants was made via an anonymous form (for the written consent, the preferred modality to express themselves, and the contact information of the principal researcher in case the women needed it). In total, 21 participants finally answered the questions, by video call (*n* = 3), via an online form (*n* = 17), or via email (*n* = 1) according to their preferences. These options were provided to make the study as inclusive as possible. The video call responses lasted an hour and were transcribed and incorporated into the database. The online form could take 45 min to complete.

Video call responses were transcribed verbatim, along with the written responses received online, following the thematic analysis model (Braun and Clarke, 2006): (i) familiarization with the data; (ii) initial coding and extracts of interest; (iii) export to Taguette software (Rampin and Rampin, 2021), an open-source qualitative research tool; (iv) identification of several patterns in the responses, corresponding to two major related categories and four and two subcategories; (v) a review of the analysis, with 90% agreement among researchers; and (vi) organization of the participants' narrative, with verbatim quotations, so that the reader can better understand the analysis.

## 3 Results

The data analysis produced two themes, the first with four sub-themes and the second with two sub-themes. Each sub-theme is discussed along with illustrative quotes from the participants (pseudonym). The concept “autistic woman” is specified as general but may refer to the autistic daughter, sister, or granddaughter, regarding the relative referred to.

### 3.1 Theme 1. Reaction to the diagnosis

From the experiences of how family and friends reacted when autistic women 'came out of the closet', four reaction sub-themes emerged: denial, dismissiveness, disbelief, and finally—the reactions encompassed mainly by friendships—support.

#### 3.1.1 Sub-theme 1.1. Denial

Most families denied the diagnosis when told by the women:

*My family denied it. They said that I didn't look like one, even that my problem was behavioral* (Laura); *My maternal family didn't believe me, they thought I had become obsessed with the subject because of my child, who is also autistic* (Melissa); *My grandmother didn't believe it, as she related autism with more well-known difficulties or with being non-verbal* (Penelope); *Her reactions covered a spectrum. We went from ‘everything fits' to 'no, you can't be autistic because...'* (Ines); or *You're not Asperger's because you're not like Adrian* (Fabiola).

Some women even remarked that their relatives concealed the diagnosis: *It was not understood, and it was concealed* (Elena); or they responded in a hostile way that it was impossible.

There were no accounts of denial of the diagnosis by any friend.

#### 3.1.2 Sub-theme 1.2. Dismissiveness

Other family members reacted dismissively when told about it, remarking that *they completely ignored it* (Anna) or that *the few [people in my family] whom I told were indifferent or skeptical* (Jana); *My brother didn't even answer, neither did his children. My cousins weren't interested* (Olivia).

#### 3.1.3 Sub-theme 1.3. Disbelief

Some women highlighted that, more than denying diagnosis, their condition was questioned, with comments among family members such as *We all have some trait that you describe and we don't say that we have Asperger*'s *because of that* (Chloe), although this disbelief was also hinted at outside the family: *In general, people do not believe it. And if they believe it, they treat you as if you were made of glass* (Valeria).

This kind of disbelief was also seen among educational or medical professionals: *You can't be autistic because you make eye contact* (Anna, recounting an experience with a psychologist); or *When I told a teacher I trusted, she, unfortunately, told me that she didn't see me as Asperger's. I was very sad* (Bruna).

Regarding friendship, one autistic woman reported the following: *Some of my friends did not believe me* (Jana).

#### 3.1.4 Sub-theme 1.4. Support

Importantly, no autistic woman reported her family reacting assertively to her diagnosis at the time of disclosure. However, many friends reacted by listening and supporting their friend when they were told but, above all, and as will be seen in the second theme, after processing that their friends were autistic: *The few friends I have told have reacted normally, they simply listened to me. My friend Luna was there throughout the whole process* (Anna); or *I think I've hardly told anyone. My friend Clarita is the person I've talked to about it the most, she took it well and we talked for hours* (Keren).

In this section, it should be noted how some initiated their diagnosis encouraged by their partners and friends, who believed and supported them at all times: *The first person who suggested it to me was my partner* (Chloe); *A friend helped me start the diagnostic process* (Penelope); *I told my partner and my friends first, they are on the spectrum too [...] My partner, his son, and I read my report together, they recognized me in everything* (Olivia).

### 3.2 Theme 2. Interest and support after the diagnosis

The second theme highlights the help received by autistic women once “some time” had elapsed after the disclosure (mostly between 6 months and 4 years).

#### 3.2.1 Sub-theme 2.1. Interest in autism after the diagnosis

After the initial situation where many women faced dismissiveness or denial of their diagnosis by their relatives, some observed improvements in certain family members*: [Of my family], who had an interest and documented themselves identified me in several aspects and understood me better* (Laura).

Nevertheless, most participants indicated that negative reactions after the diagnosis continued, weakening—even further in many cases—their relationship to the point of saying that *right now I don't have a family* (Daniela).

On the contrary, regarding friendships, after a while, autistic women expressed that friends were fundamental pillars in the process of understanding what was happening to them and supported them in everything: *My friend Luna has helped me a lot in passing me and telling me things, information about autism in women... Asking how I'm doing..*. (Anna); or even autistic women themselves were the support for their friends: *I have a group of friends with the same characteristics, several have followed in my footsteps and are evaluating the possibility of being autistic, some already confirmed* (Olivia); *As a result of my diagnosis, my friend is processing that she is also autistic* (Anna).

#### 3.2.2 Sub-theme 2.2. Help with needs and difficulties after the diagnosis

After a time, it is evident that many of them highlight the help of the mother figure when they need it, expressing that they support them at all times: *Above all, it's my mother who shows me the most empathy* (Sienna); [...] *on the other hand, my mother understands it completely but she doesn't live with me, so sometimes I feel misunderstood* (Teresa); *my mother sees my qualities* (Nora).

The sibling figure is contradictory, however, positive reactions do occur*: my brother, although he is two years younger, always gives me good advice [...]; he particularly helps me with messages when I don't understand the intention* (Teresa); although sometimes they do not know how to act: *my sister does not always take into account my difficulties or sensory sensitivities* (Penelope); *they often fail by giving me some kind of containment (e.g., sensory)* (Sienna); or even *my siblings don't support me at all, they still don't believe it, or act as if I haven't told them* (Anna).

It is striking that the majority highlighted that, on their father's side, they feel misunderstood or that he fails them when they need him, even though some of the fathers are autistic themselves: *My father does not support me. He always brings out the negative side of everything, although he also has autism spectrum disorder (ASD) (more severe than me) and well, it's understandable* (Valeria); *Because I live with my father, who doesn't understand much about the subject, I feel that he doesn't see the issue of my autism as so real* (Teresa).

Furthermore, many remarked that they did not need that help and that they could deal with their own problems or had learnt strategies (of camouflage) to solve them: *My family has enough things going on without them having to worry about what is difficult for me or not* (Valeria); *or I try to learn from what I see, without asking anyone* (Sienna); *I always ask my psychologist for help or I sort it out* (Valeria); *I am an independent adult and I have no relationship with my family. I look for what I need by myself* (Daniela).

Regarding friendships, and after “some time” had passed to assimilate their diagnosis, autistic women emphasized that their friends and partners were fundamental support axes for their needs and difficulties: *When I need help, I ask my friends* (Daniela); *My husband helps me progress* (Laura).

However, above all, after the diagnosis, they attended associations where they met other autistic women, which is where they began to feel less lost and to identify and accompany each other: *It helps me see that I am not alone and makes me have relationships with other people* (Penelope); *The association helps me be with people like me, I feel at ease and they help me when I have a problem* (Raquel); *I am the president of an association of self-represented autistic women. We defend our rights and we help each other* (Elena); *I am the president of one and a team member of another, and it helps me raise awareness and stop the condition from being a social taboo* (Melissa).

One woman is a speaker who explains autism from the first-person perspective, which not only unites her with many associations but also makes her feel valued: *I am linked to many associations since I am a speaker. I think they really value my view of things. They help me because they open that window that I need to better understand the environment every day* (Valeria).

Another woman highlights the importance of meeting more autistic women and how they make her feel part of a family: *I participate in WhatsApp groups with other women on the spectrum and we support each other, this group has been my lifeline because they understand me, I feel accompanied, understood, I thus found a family, my ASD family* (Teresa).

## 4 Discussion

This study gives voice to autistic women to better understand their experiences of receiving a diagnosis and conveying it to their family and friends. The first theme offers some hints to help understand why their family nucleus does not believe them or denies it. This denial may be because they were not present during the process and cannot understand a diagnosis in adulthood that had always been there. However, autistic women began their diagnosis encouraged by their partners and friends, who believed in and supported them at all times. The second theme, which retrieves information sometime after diagnosis, portrays the mother's redemption, who finally seeks to understand her daughter. In both themes, friendships (including the partner) are key to helping and supporting them from the beginning.

Thus, in the first theme, we find a range of reactions from family members from denying the diagnosis to exhibiting dismissiveness toward knowing what is happening with their daughter. Additionally, dismissiveness was a common negative reaction reported here and in the study by Huang et al. (2022), although it was not addressed in previous publications. They also reported disbelief, highlighting how the issue of gender influences the reactions of the people closest to them since family members are familiar with the characteristics of men and compare them with those of women (Garcia-Molina, 2022). At first, it could be believed that the family received the diagnosis as if in 'mourning' and, as in the first theme, they deny it; however, this same negative reaction continues later on, as observed in the second theme, further weakening their relationship. These two themes shed light on the question of what happens when the diagnosis is received in adulthood because when received in childhood, although they may see their expectations diminished, parents accept the diagnosis, even emphasizing and understanding them or strengthening their relationship (mother-child) (Schwichtenberg et al., 2019). This type of bond also occurs between autistic mothers and their autistic children (Crane et al., 2021). However, friends react assertively, listening and supporting their friends, including initiating the process encouraged by them and their partners. This finding is in agreement with recent studies on the friendships of autistic women, with whom they would have an intimate relationship and would be there for them, including helping them to deal with their different day-to-day difficulties (Dean et al., 2014; Sedgewick et al., 2019; Garcia-Molina, 2022).

In the second theme, the incomprehension and dismissiveness of their relatives in asking them about their diagnosis, helping them, or knowing what is happening to them stand out. A detail emerging from the vast majority of the stories is that their mothers accept and support them, while their fathers—who, surprisingly, are sometimes also diagnosed with autism—do not understand or help them. Outside their family, it is in nuclei, such as associations, where they will find psychological assistance, friendships, and partners for the desired help and understanding (Crane et al., 2021; Dugdale et al., 2021). It is notable how they can connect with the community of autistic women through associations and thus find their chosen *family* who understands them (Tan, 2018).

In conclusion, this study highlights the incomprehension of family members and the helplessness of autistic women when disclosing their diagnoses as well as the lack of subsequent help, contrasting with, on the same level, the selfless help of friends (some of whom are also in the process of diagnosis) and the autistic community. This preliminary study, while acknowledging its limitations as such, seeks to advance our understanding of autism from a gender perspective and to stop it from being a utopian reality that cases like those of the women included in this study be diagnosed at a young age. We urge that qualitative research that gives a voice to autistic women should be continued for greater dissemination. Additionally, we aim to contribute toward creating a more tolerant society for all autistic individuals. Overall, it should not be the case, after facing a late diagnosis, where autistic women also have to fight for society to believe them.

## Data availability statement

The original contributions presented in the study are included in the article, further inquiries can be directed to the corresponding author.

## Ethics statement

The studies involving humans were approved by Ethics Committee from Universitat Jaume I. The studies were conducted in accordance with the local legislation and institutional requirements. The participants provided their written informed consent to participate in this study. Written informed consent was obtained from the individual(s) for the publication of any potentially identifiable images or data included in this article.

## Author contributions

IG-M: Conceptualization, Formal analysis, Funding acquisition, Investigation, Methodology, Writing – original draft, Writing – review & editing.
